# Supplementary material for: Liposome-Mediated Gene Transfer in Differentiated HepaRG™ Cells: Expression of Liver Specific Functions and Application to the Cytochrome P450 2D6 Expression
Source: Cells. 2022 Dec 2;11(23):3904. doi: 10.3390/cells11233904 (PMC9737581; doi:10.3390/cells11233904)
Supplement: Supplementary file 1 [file cells-11-03904-s001.zip › cells-1980185-supplementary.pdf]

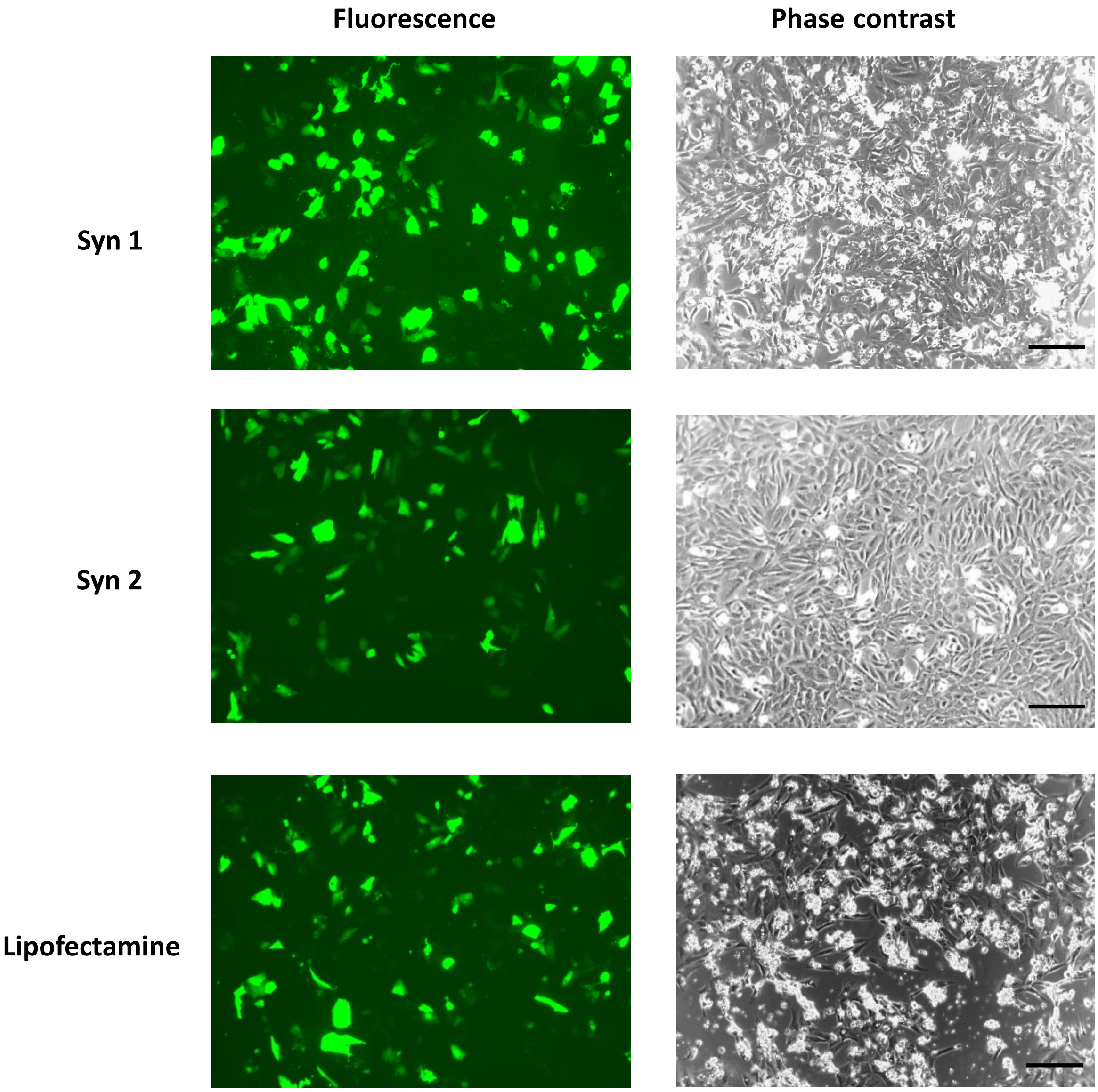

Supporting Information S1. Representative images of GFP positives progenitor HepaRG™ cells at day 2 following lipofection by the Syn 1, Syn 2 and Lipofectamine 3000 transfection reagents prior detachment of cells for flow cytometry analysis. Bars: 100 μm.

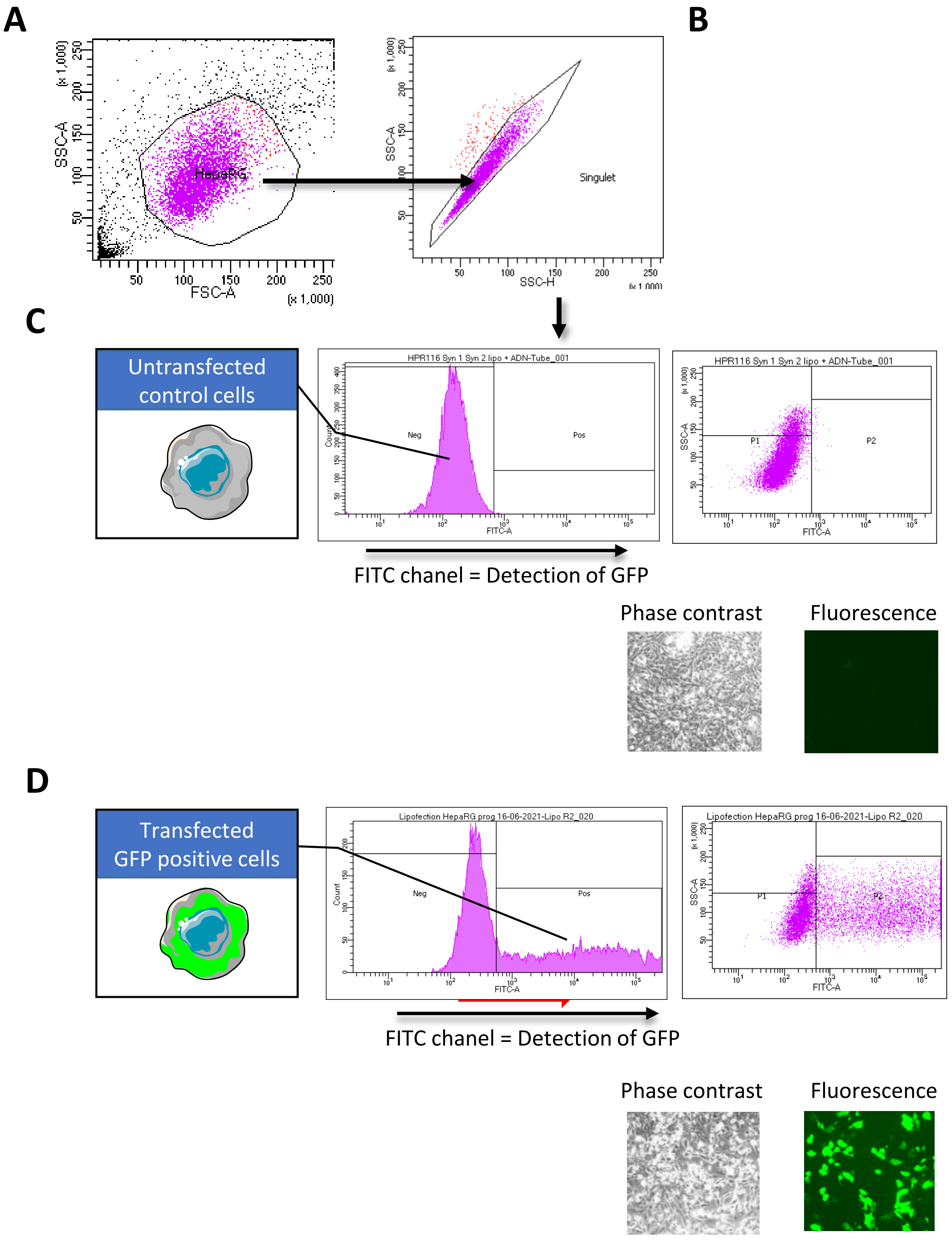

Supporting Information S2. The events (cells) analyzed by FACS were gated using dot plots with the Side SCatter (SSC, granularity on y axis) and Forward SCatter (FSC, size on x axis) parameters (**A**). Then, events from this first cell population were further gated using the SSC-Height versus SSC-Area dot plot to isolate the single cells (**B**). From the single cell population, fluorescence intensities were analyzed (histogram: fluorescence on x axis versus cell count on y axis ; dot plot: fluorescence on x axis versus SSC-Area on y axis) in cells that were not transfected to define the "background" fluorescence also called auto-fluorescence of negative cells (Neg) (**C**). In non-transfected control cells, no GFP^+ cells were detected and the autofluorescence value (MFI) was arbitrarily set as 105 arbitrary units. Then, the cells transfected using plasmid encoding the GFP were analyzed (**D**), which defined the GFP positive cells (Pos). The values of MFI presented in this work represent the overall fluorescence of all the single GFP^+ cells (Pos). After acquiring fluorescence signals of ~10^4 cells, percentage of GFP^+ cells, fluorescence intensities of single cells were obtained from the FACSDiva and flowLogic softwares for transfected cells. Prior detachment of cells for flow cytometry analysis, GFP positive (GFP^+) cells can be visualized by fluorescence microscopy.

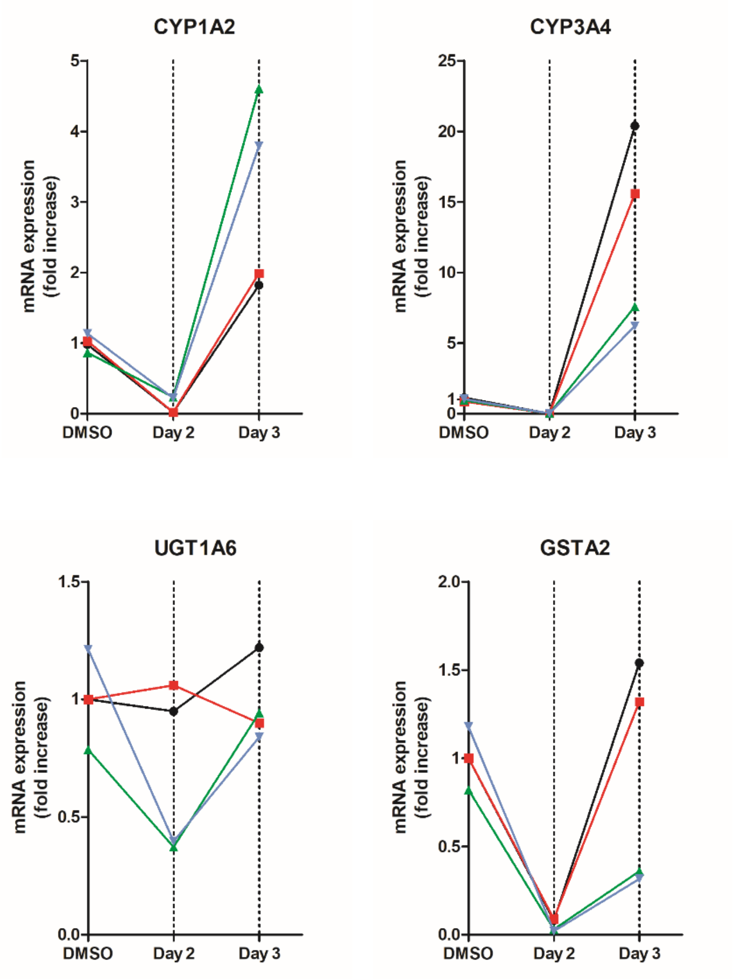

Supporting Information S3. Variabilities in data from Figure 4 were observed, which may be explained by the fact that the experiments were performed over a long period of time with distinct lots of HepaRG cells at different passages and using different batches of fetal calf serum. However, all independent experiments showed the same pattern in gene expressions with a significant decrease in some hepatocyte specific functions such as albumin, CYP1A2, 2E1, 3A4 and other phase II genes. As example, we provide in Supporting Information 3, the gene expression profiles of CYP1A2, CYP3A4, UGT1A6 and GTSA2 in 4 independent experiments (n=4) showing similar and significant changes in expressions for CYP1A2, CYP3A4 in the 3 experimental conditions while the patterns of expressions showed no significant differences for UGT1A (fold changes < 0.5).
